# Supplementary material for: Two de novo GluN2B mutations affect multiple NMDAR-functions and instigate severe pediatric encephalopathy
Source: eLife. 2021 Jul 2;10:e67555. doi: 10.7554/eLife.67555 (PMC8260228; doi:10.7554/eLife.67555)
Supplement: Source code 1. [file elife-67555-code1.zip › codeforkellnerdeltadeltaG.docx]

strori = "DDHLSIVTLEEAPFVIVESVDPLSGTCMRNTVPCQKRGYIKKCCKGFCIDILKKISKSVKFTYDLYLVTNGKHGKKINGTWNGMIGEVVMKRAYMAVGSLTINEERSEVVDFSVPFIETGISVMVSAAFMIQEEYVDQVSGLSDKKFQRPNDFSPPFRFGTVPNGSTERNIRNNYAEMHAYMGKFNQRGVDDALLSLKTGKLDAFIYDAAVLNYMAGRDEGCKLVTIGSGKVFASTGYGIAIQKDSGWKRQVDLAILQLFGDGEMEELEALWLTGICHE";

strsplit = num2cell(char(strori));

pos = [403:1:439 451:1:539 651:1:803];

aminoacids = ["H","Q","P","R","L","D","E","A","G","V","Y","S","C","W","F","N","K","T","I","M"];

for i = 1:length(strsplit)

for k = 1:length(aminoacids)

if strsplit(i) == aminoacids(k)

continue

end

finalstr((i-1)*20+k) = append(strsplit(i),int2str(pos(i)),aminoacids(k));

end

end
