# Supplementary material for: Two de novo GluN2B mutations affect multiple NMDAR-functions and instigate severe pediatric encephalopathy
Source: eLife. 2021 Jul 2;10:e67555. doi: 10.7554/eLife.67555 (PMC8260228; doi:10.7554/eLife.67555)
Supplement: Supplementary file 1. — Description of output, functional validation and clinical pictures for reported GRIN2B variants. ATD- amino terminal domain; LBD- ligand binding domain; CTD- carboxy terminal domain; GoF and LoF- gain and loss-of-function, respectively. [file elife-67555-supp1.docx]

| **Published functionally validated GluN2B mutations** | | | | | | |
| --- | --- | --- | --- | --- | --- | --- |
| No. | Residue | domain | Output | Functional validation | Phenotype | Ref |
| 1 | S34G  lnfsX25 | ATD | LOF | no current recorded | ASD, ID | doi:10.1136/jmedgenet-2016-104509 |
| 2 | Q180* | ATD | LOF | no current recorded | ID, fSz | doi:10.1136/jmedgenet-2016-104509 |
| 3 | E413G | LBD (S1) | LOF | Decreased: glutamate affinity, τ_off_ , peak amplitude, expression levels | ID, hypotonia | doi.org/10.1016/j.ajhg.2016.10.002 |
| 4 | C436R | LBD (S1) | LOF | Decreased: expression levels  Did not responded to glutamate application (<50nA with 1mM glu), | ID, Epi | doi.org/10.1016/j.ajhg.2016.10.002 |
| 5 | C456Y | LBD (S1) | LOF | Increased: glutamate affinity  Decreased: glycine affinity, peak amplitude, expression levels | ID | doi.org/10.1016/j.ajhg.2016.10.002 |
| 5 | C456Y (animal model) | LBD (S1) | LOF | Increased: P_open_  Decreased: amplitude (>1% of wt), proton inhibition, spermine potentiation, glycine affinity, NMDAR/AMPA receptor mediated evoked excitatory postsynaptic currents (EPSCs), NMDA dependent EPSCs τ_off_ , NMDAR-dependent LTD |  | doi.org/10.1371/journal.pbio.3000717 |
| 6 | C461F | LBD (S1) | LOF | Increased: glycine affinity  Decreased: glutamate affinity, τ_off_, peak amplitude, expression levels, eEPSC τ_off_ | ID, Epi | doi.org/10.1016/j.ajhg.2016.10.002  DOI: 10.1038/s41467-018-02927-4 |
| 7 | R519* | LBD (S1) | LOF | Decreased: amplitude, surface expression | Mild ID | doi.org/10.1093/hmg/ddaa220 |
| 8 | R540H | LBD (S1) | GOF | Increased: glutamate affinity, glycine affinity, τ_off_, calcium permeability  Decreased: expression levels, Mg^2+^ inhibition | ID, Epi | doi.org/10.1016/j.ajhg.2016.10.002  DOI: 10.1002/ana.24073 |
| 9 | S541R | Linker (2) | LOF | Decreased: Glutamate affinity, Glycine affinity, | Severe ID, ASD, gSz, dystMD | doi:10.1136/jmedgenet-2016-104509 |
| 10 | P553L | Linker (2) | LOF | Increased: t_off_ (HEK293)  Decreased: glutamate affinity, eEPSC peak amplitude, NMDAR-mediated EPSCs τ_off_ , surface/total expression | Severe ID | DOI: 10.1038/s41467-018-02927-4  DOI:10.1371/journal.pgen.1006536  doi: 10.3389/fnmol.2018.00110 |
| 11 | P553T | Linker (2) | LOF | Decreased: glutamate affinity, amplitude, conductance, τ_off_ , peak P_open_ , EPSC amplitude | ID, Rett-like syndrome | DOI: 10.1126/scisignal.aaw0936 |
| 12 | V558I | M1 | LOF | Increased: τ_off_  Decreased: glutamate affinity, P_open_ | Moderate ID | doi: 10.3389/fnmol.2018.00110  doi:10.1136/jmedgenet-2016-104509 |
| 13 | W559* | M1 | LOF | no current recorded | ASD, ID | doi:10.1136/jmedgenet-2016-104509 |
| 14 | W607C | M2 | LOF | Decreased: glutamate affinity, glycine affinity, Mg^2+^ inhibition, proton inhibition, whole cell & single-channel amplitude, P_open_, mean open time, surface/total expression. | DD, ID, dysmorphic features | DOI: 10.1002/humu.23895  doi: 10.3389/fnmol.2018.00110 |
| 15 | G611V | M2 | GOF | Decreased: Mg^2+^ sensitivity (N.E), amplitude, P_open_, mean open time, memantine inhibition | Epi, ID, dysmorphic features  Severe ID, gSz, MC | DOI: 10.1002/humu.23895  doi:10.1136/jmedgenet-2016-104509 |
| 15 | N615I | M2 | GOF | Increased: glycine affinity, memantine inhibition  Decreased: glutamate affinity, Mg^2+^ inhibition, P_open_, Ca^2+^ permeability, Mg^2^ block of NMDAR-mediated EPSCs, whole cell & single-channel amplitude | ASD, Epi, hypotonia, ID, WS | DOI: 10.1038/s41467-018-02927-4  doi: 10.3389/fnmol.2018.00110  DOI: 10.1002/humu.23895  doi:10.1136/jmedgenet-2016-104509 |
| 16 | N615K | M2 | GOF | Increased: glycine affinity  Decreased: Mg^2+^ sensitivity, amplitude, P_open,_ | DD, ID | DOI: 10.1002/humu.23895 |
| 17 | N616K | M2 | GOF | Increased: glycine affinity  Decreased: Mg^2+^ sensitivity, P_open_ | Epi, ID, dysmorphic features, hypertonia | DOI: 10.1002/humu.23895 |
| 18 | V618G | M2 | LOF | Decreased: Mg^2+^ inhibition, proton inhibition, memantine inhibition, whole cell & single-channel amplitude, mean open time, calcium permeability, Mg^2^ block of NMDAR-mediated EPSCs | Epi, ID, hypotonia, WS, Epileptic encephalopathy | DOI: 10.1002/humu.23895  DOI: 10.1038/s41467-018-02927-4  doi: 10.3389/fnmol.2018.00110 |
| 19 | V620M | M2 | GOF | Increased: τ_off_, P_open_  Decreased: Mg^2+^ inhibition, proton inhibition, calcium permeability | DD, ID, hypotonia | DOI: 10.1002/humu.23895 |
| 20 | S628F | M3 | LOF | Decreased: surface/total expression  Did not responded to 1 mM glutamate application | ID, DD, Epileptic encephalopathy | doi: 10.3389/fnmol.2018.00110 |
| 21 | A636P | M3 | LOF | no current recorded | Mild ID | doi:10.1136/jmedgenet-2016-104509 |
| 22 | I655F | M3 | GOF | Decreased: glutamate affinity, Mg^2+^ inhibition, proton inhibition | Severe ID, ES, gSz, MCD, MC, CVI | doi:10.1136/jmedgenet-2016-104509 |
| 23 | Q656* | Linker (5) | LOF | no current recorded | moderate ID, fSz | doi:10.1136/jmedgenet-2016-104509 |
| 24 | E657G | Linker (5) | LOF | Increased: glutamate affinity  Decreased: glycine affinity, amplitude | ID, DD | doi: 10.3389/fnmol.2018.00110 |
| 25 | R682C | LBD (S2) | GOF | Increased: glutamate affinity, glycine affinity, τ_off_  Decreased: total expression | ID, Epi | doi.org/10.1016/j.ajhg.2016.10.002  doi:10.1038/ng.677 |
| 26 | G689C | LBD (S2) | LOF | Decreased: glutamate affinity, glycine affinity, surface expression, proton inhibition, spermine potentiation, NMDAR dependent EPSCs τ_off_. | Epi, DD, ID, hypertonia, dyskMD | current paper |
| 27 | G689S | LBD (S2) | LOF | Decreased: glutamate affinity, proton inhibition, spermine potentiation, apparent P_open_, NMDA dependent EPSCs τ_off._ | DD, ID, hyportonia, dyskMD, Strabismus | current paper |
| 28 | R696H | LBD (S2) | GOF | Increased: glutamate affinity, τ_off_  Decreased: amplitude, expression levels | ID | doi.org/10.1016/j.ajhg.2016.10.002 |
| 29 | M706V | LBD (S2) | Neither | Unknown functional problems (glu, gly, Mg^2+^ & proton inhibition were unaffected) | Severe ID, fSz | doi:10.1136/jmedgenet-2016-104509 |
| 30 | G724* | LBD (S2) | LOF | Decreased: No NMDA dependent currents upon calcium imaging, surface expression, dendrite development (number and length of dendrite segments). | ASD, ID | doi:10.1242/jcs.232892 |
| 31 | D786Mfs*23 | LBD (S2) | LOF | Decreased: amplitude, surface expression | Mild ID, hypotonia, ASD traits, digestive problems, abnormal EEG during sleep without seizures | doi.org/10.1093/hmg/ddaa220 |
| 32 | S810R | Linker (6) | GOF | Increased: glutamate affinity, glycine affinity  Decreased: proton inhibition, memantine inhibition | Severe ID, fSz, MCD, MC | doi:10.1136/jmedgenet-2016-104509 |
| 33 | M818T | M4 | GOF | Increased: glutamate affinity, glycine affinity  Decreased: proton inhibition, memantine inhibition | DD, ES, fSz, gSz, CVI | doi:10.1136/jmedgenet-2016-104509 |
| 34 | A819T | M4 | GOF | Increased: glutamate affinity, glycine affinity  Decreased: proton inhibition, memantine inhibition | DD, ID, Sz | doi:10.1136/jmedgenet-2016-104509 |
| 33 | G820A | M4 | LOF | Decreased: amplitude, τ_off_, P_open_ | ID, ES, dyskMD, DD, ASD, GVL | DOI: 10.1038/s41467-018-06145-w  doi:10.1136/jmedgenet-2016-104509  doi: 10.3389/fnmol.2018.00110 |
| 34 | G820E | M4 | LOF | No current recorded.  increased: surface/total expression | ID, DD, DMD, ES, GVL, ASD | doi:10.1136/jmedgenet-2016-104509  DOI: 10.1038/s41467-018-06145-w  doi: 10.3389/fnmol.2018.00110 |
| 35 | M824R | M4 | LOF | no current recorded | ID, DD, microcephaly, Rett-like picture, Epi activity on EEG | doi:10.1136/jmedgenet-2016-104509  doi: 10.3389/fnmol.2018.00110 |
| 36 | L825V | M4 | LOF | Decreased: P_open_ | ASD, ID | doi: 10.3389/fnmol.2018.00110  DOI: 10.1038/s41467-018-06145-w  doi:10.1136/jmedgenet-2016-104509 |
| 37 | G826E | M4 | GOF | Increased: τ_off_ | ID | DOI: 10.1038/s41467-018-06145-w |
| 38 | E839* | CTD | LOF | Decreased: amplitude, surface expression | Mild ID, abnormal EEG, ECSWS | doi.org/10.1093/hmg/ddaa220 |
| 39 | S1413L (RAT)  S1415L  (Human) | CTD | LOF | Decreased: surface expression, Binding to MGUKs (binding to PSD-95), NMDAR-mediated EPSCs amplitude, dendritic spines | ASD | DOI:10.1523/JNEUROSCI.0827-16.2017 |
| 30 | L1422F (RAT)  L1424L (Human) | CTD | LOF | Decreased: Binding to MGUKs (binding to PSD-95), glutamate affinity. | SCZ | DOI:10.1523/JNEUROSCI.0827-16.2017 |
| 41 | S1450F (RAT)  S1452F (Human) | CTD | LOF | Decreased: Binding to MGUKs (binding to PSD-95)  Increased: glycine affinity. | SCZ | DOI:10.1523/JNEUROSCI.0827-16.2017 |

ASD, autism spectrum disorder; ATD, amino-terminal domain; CTD, carboxy-terminal domain; CVI, cortical visual impairment; DD, developmental delay; dyskMD, dyskinetic movement disorder; dystMD, dystonic movement disorder; ECSWS, continuous spike-and-wave during slow wave

sleep; ES, epileptic spasms; fSz, focal seizures; gSz, generalised seizures; GVL, generalised cerebral volume loss; ID, intellectual disability; MC, microcephaly; MCD, malformation of cortical development; M1–M4, transmembrane domain; NA, not available; schizophrenia, SCZ; Sz, seizures (not further classified); S1, S2, ligand-binding domain; WS, West Syndrome.
