# Supplementary material for: Two de novo GluN2B mutations affect multiple NMDAR-functions and instigate severe pediatric encephalopathy
Source: eLife. 2021 Jul 2;10:e67555. doi: 10.7554/eLife.67555 (PMC8260228; doi:10.7554/eLife.67555)

Kellner and Berlin. Supplementary File 3

a

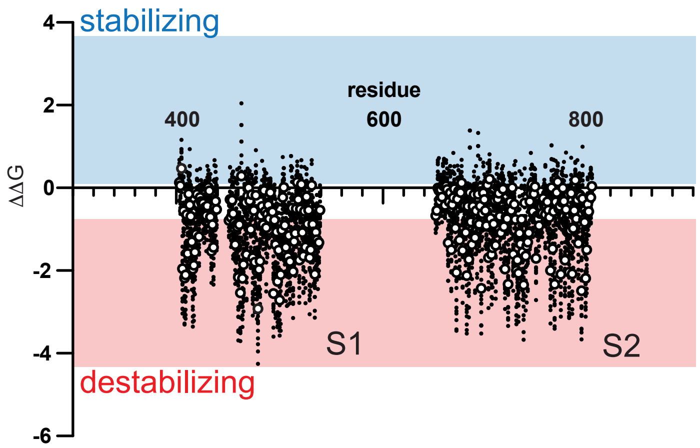

b

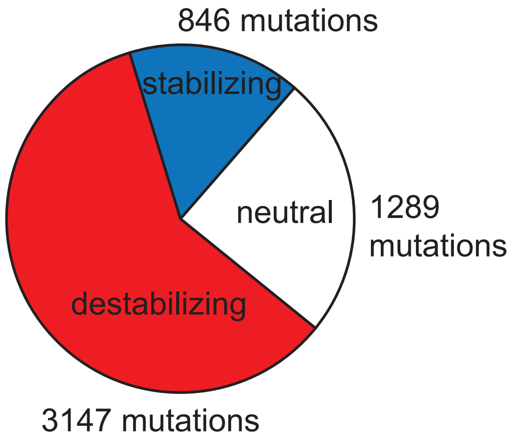

c

Mutation	Functional output	EC_{50} (μM)	ΔΔG	Comment
C436R	LOF	1	-1.505	
R696H	GOF	0.33	-1.486	
G689C	LOF	1100	-1.42	
G689S	LOF	1800	-1.355	
M706V	neither	1.5	-1.15	
E413G	LOF	79	-1.054	
C461F	LOF	169	-0.86	
E657G	LOF	0.7	-0.828	
C456Y	LOF	0.39	-0.804	
C456Y	LOF		-0.804	
R682C	GOF	0.91	-0.703	
I655F	GOF	5.6	-0.641	
Q656	LOF		-	stop
G724	LOF		-	stop
R519	LOF		-	stop
D786M	LOF		-	stop

d

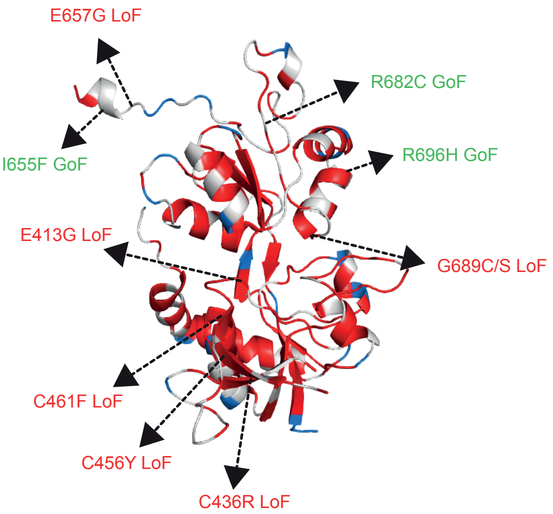

e

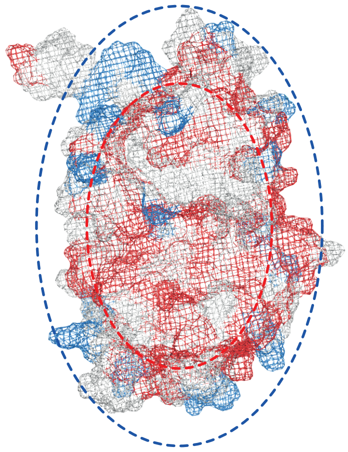

f

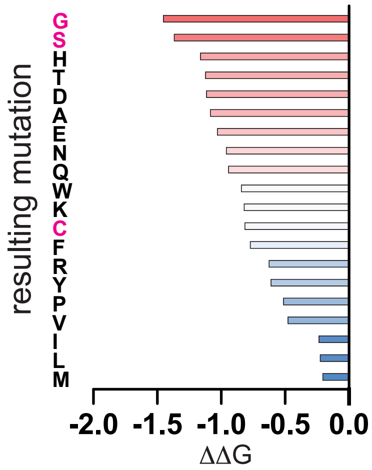

Supplement: Supplementary file 3. — a. ΔΔG values for 5282 substitutions in the S1/S2 segments (positions 403–539, 651–802) of GluN2B. Stabilizing mutations are represented by ΔΔG>0 (blue box), whereas destabilizing (ΔΔG< −0.5) are marked by red box. b. Most mutations within LBD are destabilizing (~60%; red), with the smallest fraction represented by stabilizing mutations (~16%; blue). Mutations with minor or no effect are noted by white color. c. List of 18 validated LBD mutations in GluN2B and their estimated ΔΔG. Point mutations resulting in stop codons are noted as stop. d. Overlay between mutations shown in c. (dashed arrows) and mean ΔΔG; calculated for each residue by averaging all its possible substitutions for that residue. Red- destabilizing, blue-stabilizing; gray-neutral effect. e. Surface view of the LBD shows that stabilizing residues (blue, taken from d) are located on the outer surface of the LBD. f. Potential effect of all amino acids if these were substituted anywhere in the LBD. This consists of averaging the effect of each substitution across the entire LBD. Analysis highlights glycine (G) and serine (S) as the most destabilizing mutations, whereas methionine (M) as the most stabilizing. [file elife-67555-supp3.pdf]
